# Toward value in healthcare: opportunities and challenges for implementing Value-Based Healthcare in North Macedonia

**DOI:** 10.3389/fpubh.2025.1646870

**Published:** 2025-10-15

**Authors:** Aleksandra Grozdanova, Evgenija Mihajloska, Zorica Naumovska, Marija Staninova Stojovska, Zoran Sterjev, Aleksandar Dimkovski, Katerina Anchevska Netkovska

**Affiliations:** Faculty of Pharmacy, Ss Cyril and Methodius University in Skopje, Skopje, North Macedonia

**Keywords:** Value-Based Healthcare (VBHC), North Macedonia, health system reforms, health system sustainability, patient centered

## Abstract

**Objective:**

European healthcare systems face increasing pressures from ageing populations, chronic disease burdens, and rising costs. North Macedonia experiences similar challenges, including workforce shortages, limited economic growth, and persistent health inequalities. Although reforms such as the Diagnosis-Related Group payment system and the development of an e-health platform represent progress, major gaps remain: low public funding, high out-of-pocket spending, weak primary care, and poor data integration. This review analyses Value-Based Healthcare implementation in selected European countries and assesses the current state of the Macedonian health system to identify challenges and opportunities for Value-Based Healthcare.

**Methods:**

A two-step narrative approach was applied. Peer-reviewed literature on Value-Based Healthcare implementation in European countries was retrieved from PubMed, Scopus, and Web of Science, complemented by case studies and EIT Health handbook. To evaluate North Macedonia, policy documents and reports were reviewed from the World Health Organization, the European Observatory on Health Systems and Policies, the European Commission, and national authorities, including the Health Insurance Fund, the Institute of Public Health, and the Ministry of Health. Evidence was synthesized thematically across financing, digital infrastructure, outcome measurement, and governance.

**Results:**

Examples from Spain, the Netherlands, and Germany demonstrate the benefits of outcome measurement, integrated care delivery, and innovative payment models. Experiences from Bulgaria and Slovenia, with comparable health systems, provide additional lessons.

**Conclusion:**

Value-Based Healthcare implementation in North Macedonia will require greater public investment, stronger primary care, standardised outcome measurement, and robust digital infrastructure. Prioritising value-driven, patient-centered reforms could strengthen performance, equity, and sustainability.

## 1 Introduction

In recent years, healthcare systems across Europe have faced numerous challenges, including an aging population and a rising prevalence of chronic diseases that require prolonged and costly treatments. Additionally, the healthcare sector is dealing with workforce shortages, increasing treatment costs, and unstable political environments—all of which threaten the sustainability and equity of healthcare services (1). In response to rising drug prices, which pose a significant challenge to maintaining access to innovative therapies, European policymakers are increasingly implementing cost-containment measures. These include value-based evidence and comparative effectiveness data through health technology assessments (HTAs) (2). Such changes, alongside rapid technological advancements and unexpected events such as economic downturns or pandemics, underscore the urgent need for more resilient and efficient health systems. In this context, the World Health Organization (WHO) publication *Building on Value-Based Health Care: Towards a Health System Perspective* emphasizes that health systems worldwide must optimize the value derived from their existing resources (3).

The concept of Value-Based Healthcare (VBHC) is best understood as a continuum shaped by a century-long quality movement and evolving global health priorities (4). In 2000, the WHO identified three universal health system goals: improving population health, meeting people’s expectations, and ensuring financial protection. Porter and Teisberg later in 2006 defined value as the health outcomes per dollar spent, while the Institute for Healthcare Improvement expanded this vision through the Triple Aim, emphasizing better population health, enhanced patient experience, and lower costs of care (4). More recently, the Expert Panel on Effective Ways of Investing in Health proposed a broader European perspective that defines VBHC as a comprehensive concept built on four value pillars: appropriate care to achieve each patient’s personal goals (personal value), achievement of best possible outcomes with available resources (technical value), equitable resource distribution across all patient groups (allocative value) and contribution of healthcare to social participation and connectedness societal value (5).

According to Porter, the strategic agenda for achieving a high-value healthcare system has six components: organizing care into Integrated Practice Units dedicated to specific conditions or patient groups; measuring outcomes and costs for every patient in a standardized and transparent way, with a focus on measuring the cost of care and the outcomes that matter to patients; adopting bundled payments: (a) payment approach that covers the full care cycle for acute medical conditions, (b) the overall care for chronic conditions for a defined period, (c) primary and preventive care for a defined patient population; integrated health delivery systems that coordinate services across specialties, facilities, and levels of care to improve efficiency and continuity; expand geographic reach to extend healthcare access for more patients and developing an enabling IT platform to support outcome measurement, data exchange, and continuous improvement (6, 7).

Implementing the VBHC model presents several challenges for stakeholders across healthcare systems as they transition from traditional fee-for-service or capitation models. This transition requires systemic changes, including: the provision of appropriate tools for tracking treatment outcomes; expert analysis of health data; upgraded information systems capable of monitoring patient costs; facilitation of data sharing among providers; benchmarking; value-based payment models; and strategic change and innovation ecosystems (8). While the specifics of VBHC implementation may vary among countries, the overarching objective—to increase value for patients—remains constant (9).

## 2 Objective

The objective of this narrative review is to analyze the implementation of VBHC in selected European countries—specifically Spain, Netherlands and Germany, which are widely regarded as models of good practice, and Bulgaria and Slovenia, whose health systems share similarities with that of North Macedonia. A second objective is to assess the current state of the healthcare system in the Republic of North Macedonia, with a particular focus on identifying the key challenges that may influence the country’s capacity to adopt the VBHC model.

## 3 Methods

This narrative mini-review applied a two-step approach aligned with the study objectives. First, the implementation of VBHC in selected European countries was examined through peer-reviewed literature (2006–2025) identified in PubMed, Scopus, and Web of Science, supplemented by case studies and implementation handbook from EIT Health and other international experts. Second, the current status of the healthcare system in North Macedonia was evaluated using policy documents and reports from the WHO, the European Observatory on Health Systems and Policies, the European Commission, and national authorities, including annual reports of the Health Insurance Fund, analyses from the Institute of Public Health, and the National Health Strategy 2021–2030 issued by the Ministry of Health. Extracted evidence was synthesized thematically across four domains—financing, digital infrastructure outcome measurement, and governance—to identify systemic challenges for VBHC implementation.

## 4 Models of VBHC implementation in different European countries

In Europe, VBHC is increasingly recognized as a transformative model that fosters collaboration among healthcare organizations to deliver high-quality, patient-centered care while optimizing resource use and reducing cost (10). The core principles of VBHC focusing on outcomes that matter to patients, organizing care around medical conditions, and shifting from volume- to value-based payment systems have been successfully implemented across diverse healthcare systems, both public and private, in several EU member states (11, 12).

European health systems generally fall under one of three co-existing management models: National Health Services, Social Insurance-based Systems or Mixed Model systems-each with distinct funding methods and service delivery approaches (13). The successful adoption of VBHC across European Union member states is influenced by differences in healthcare financing models, system fragmentation, and varying levels of digitalization. Some barriers—such as fragmented care, lack of outcome measurement, inadequate patient-level costing, and fee-for-service incentives, are common across all European health systems. However, their intensity and policy solutions vary by system type (14).

Based on successful implementation examples in countries such as Spain, the Netherlands, and Germany, the European Institute of Innovation & Technology (EIT) Health published the *Implementing Value-Based Health Care: Handbook for Pioneers*, which provides actionable strategies for real-world VBHC adoption (15). The handbook highlights critical elements including patient-centered outcome measurement, data standardization, team-based care, and continuous improvement. Its implementation matrix consists of five key steps: recording outcomes and processes through data platforms; comparing results using benchmarking; rewarding performance with outcome-based incentives; improving care via collective learning cycles; and partnering internally and externally to scale innovation (15, 16).

The Diabeter Network in the Netherlands is a successful example of VBHC implementation, offering integrated, patient-centered care for individuals with Type 1 diabetes. Operating as a private, non-profit Integrated Practice Unit, Diabeter employs a multidisciplinary team responsible for the entire healthcare cycle. Its system systematically collects and analyzes standardized outcomes to support personalized treatment adjustments, resulting in improved clinical outcomes and reduced healthcare costs (17). Similarly, Germany’s Martini Klinik, a privately operated center specializing in prostate cancer, has received international acclaim for its rigorous outcome measurement practices. The clinic systematically documents treatment results not only for the clinical research but also to support continuous performance improvement among surgeons (18). In Spain and the Netherlands, the VBHC model is implemented through large hospital networks—Quirónsalud and the Netherlands Heart Network, respectively which emphasize coordinated service delivery, standardized outcome measurement, and data-driven improvement, including the use of patient-reported outcomes and experience measures (PROMs and PREMs) into clinical practice, enhancing the decision-making process with patient-reported data (19, 20).

Slovenia has initiated pilot projects in orthopedics, with the National Arthroplasty Registry at Valdoltra Orthopaedic Hospital collecting patient-reported outcomes for hip and knee replacements using validated tools like the Oxford Hip and Knee Scores and EQ-5D-5L (21). In 2023, Slovenia’s Ministry of Health introduce the National Strategy on quality and safety in Healthcare recommending the monitoring of specific patient-reported outcomes (21, 22).

In Bulgaria, the adoption of VBHC is also progressing through focused pilot projects. In rheumatology, targeted patients with inflammatory arthritis and included stakeholder training, mapping of patientcare journeys, and gap analyses to develop integrated care pathways and improve health information systems (23). In ophthalmology, pilots applying time-driven activity based-costing and patient-reported outcome measures have underscored the value of standardized data collection and continuous team education (24). Sustained training ensures that clinicians, administrators, and allied health professionals can interpret outcomes consistently, adapt to new methodologies, and embed VBHC principles into routine practice.

## 5 Overview of the healthcare system and key challenges for VBHC implementation in North Macedonia

In the Republic of North Macedonia, healthcare financing is based on the Bismarck model, in which healthcare is primarily funded through mandatory health insurance. Voluntary health insurance is also available to cover services not included in the mandatory package. The Bismarck model is a widely adopted method of healthcare financing in several developed countries, including Germany, France, the Netherlands, Belgium, Austria, Switzerland, and Luxembourg (24). North Macedonia’s healthcare system is primarily financed through social health insurance scheme managed by the Health Insurance Fund—which acts as the main purchaser of public health services. While the Health Insurance Fund oversees financial flows, the Ministry of Health plays a key role in health policy development, and the Ministry of Finance is responsible for allocating the Fund’s budget (25). The system’s primary sources of funding include salary-based insurance contributions, government budget transfers, and patient co-payments (24). In 2024, the number of insured persons covered by mandatory health insurance was 1,839,537. In 2024, the total number of insured persons decreased by 17,201 compared to 2023. The number of pensioners rose by 3,812, reaching 353.5 thousand. Meanwhile, the number of family members continued its downward trend, dropping by 20,089 (3.1%) to 637.7 thousand (26).

The health system has experienced ongoing development over time. In 2009, the Diagnosis-Related Groups (DRG) system was implemented as a case-based payment model for hospitals. Its goals were to encourage competition among healthcare providers, enable cost benchmarking, and improve the efficiency of care delivery. Beyond its role in recording and billing acute hospital services, the DRG system also serves as a funding mechanism for hospital care. It promotes the standardization of treatment procedures grounded in evidence-based medicine by employing protocols and clinical pathways tailored to each diagnosis. Additionally, it facilitates reimbursement based on the average cost of services delivered across healthcare institutions (27).

In 2019, the Ministry of Health reintroduced the National Health Accounts (NHA) within the State Statistical Office, enabling systematic monitoring of financial flows and spending by care level and disease group. Supported by WHO, this initiative strengthened national capacity for evidence based policymaking, with NHA data now published regularly for use by stakeholders (28).

In parallel with efforts to improve financial tracking, the structure of healthcare provider payments has also evolved. The Health Insurance Fund applies different payment methods according to the healthcare level: primary care providers are reimbursed via a capitation system, while secondary and tertiary care institutions receive funding through a combination of global budgets, activity-based payments, and complexity-adjusted rates determined by DRGs (25, 26). In 2024, capitation payments to general practitioners increased by 4.67% compared to 2023, while the number of general practitioners decreased by 8. Compared to the previous year, there is also a decrease in the total number of doctors in primary health care. In 2024, the total number of doctors is 2,796, a decrease of 45 doctors (26). Significant health system reforms include the 2013 Health Network, aimed at better resource planning and equitable geographic distribution of both public and private healthcare providers, and a nationwide e-health system in 2012—“My Appointment” (*Moj Termin*) system, which streamlines referrals, improves access to electronic health records, and enables monitoring of procedures and prescriptions (29). The “My Appointment” system collects robust data on a number of indicators, including financial protection, and NHA were successfully introduced in 2019, but their potential to inform policies is still underutilized. There is no systematic assessment of patients’ and providers’ satisfaction (28).

Despite the numerous reforms and improvements, North Macedonia’s healthcare system remains affected by persistent social, economic, and political challenges. One of its most critical weaknesses is the declining and insufficient allocation of gross domestic product (GDP) to health. The country spends less per capita on healthcare compared to the averages in both the WHO European Region and the European Union. In 2021, health spending represented 8.5% of GDP—close to the WHO European Region average of 8.7% but below the EU average of 9.4%. That same year, public funding covered only 54.5% of total health expenditure (28). Consequently, out-of-pocket payments were high at 41.7%, far exceeding the EU average of 15.0%, placing a significant financial burden on citizens despite their mandatory contributions to the national health insurance system (28, 29). These private expenses largely consist of co-payments for partially covered services, as well as direct payments for over-the-counter medications and services excluded from the public insurance scheme. High out-of-pocket payments costs are driven by systemic barriers such as long waiting times for diagnostics and specialist care, the migration of experienced physicians to the private sector and inadequate service quality control (29). The World Health Organization considers private health expenditures exceeding 30% of total health spending to be a critical threshold, as such high out-of-pocket costs substantially increase the risk of financial hardship and poverty among households. Recent data indicate that North Macedonia has surpassed this threshold, with private payments accounting for 41% of healthcare spending. This level is well above the internationally recommended range of 15–30%, signaling an urgent need for policy makers and health authorities to implement measures aimed at reducing the financial burden on patients and improving healthcare affordability (28, 29).

Another challenge in North Macedonia’s healthcare system is its fragmented primary care and limited scope of practice for providers, leading to overuse of secondary and tertiary care and avoidable hospital admissions (28). Fragmentation is also evident in the healthcare IT system, which suffers from incompatible data standards, poor data-sharing practices, and a lack of digital solutions (30). These issues further hinder the system’s ability to provide coordinated and quality care. The implementation of the centralized e-health platform “My Appointment” which allows insured individuals to select a physician and schedule appointments electronically, has significantly improved service accessibility and convenience. The system aggregates data from over 70 sources, including primary care providers, hospitals and clinics, offering a valuable overview of provider activity and capacity (31, 32). However, this data is not yet fully utilized for key functions such as health policy development, effective system management, or the measurement and improvement of healthcare quality. There is currently no network data connection between public and private healthcare providers, resulting in frequent gaps in patient records, as diagnostic results and treatments received in the private health sector are often not integrated into the central system. Consequently, data security, connectivity, and synchronization with the centralized system remain essential needs (33–35).

**Figure 1:**
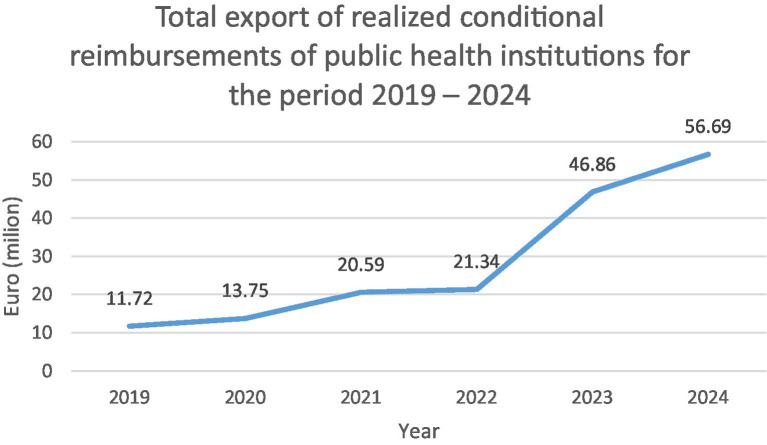
Total export of realized conditional reimbursements of public health institutions for the period 2019–2024. Source: Health Insurance Fund of the Republic of North Macedonia. Annual Report 2024. Original amounts in denars have been converted to euros for consistency.

North Macedonia’s healthcare payment system primarily relies on a fee-for-service model within a fixed annual budget, encouraging providers to increase service volume to meet financial goals. This often results in a focus on higher-paid procedures. In an effort to strengthen strategic purchasing and reduce inefficient or low-quality services, the Health Insurance Fund now allocates “conditional budgets” to public secondary and tertiary care providers, setting specific targets for the volume of services delivered. As illustrated in Figure 1, these budgets have steadily increased, reaching 56.9 million euros in 2024 across 29 public health institutions (27). This high level of expenditure highlights the urgent need for more strategic planning models to ensure efficient use of resources.

High out-of-pocket spending, rising healthcare costs, fragmented service delivery, weak primary care, and limited use of digital tools represent major challenges to the implementation of VBHC in North Macedonia. Similar to Bulgaria, which operates a mixed health system heavily reliant on private funding through voluntary insurance and out-of-pocket payments, North Macedonia faces difficulties in introducing standardized outcome-based payment models and ensuring equitable access to care. These systemic weaknesses underline the urgent need for value-based reforms to improve efficiency, strengthen patient-centered care, and build long-term system resilience.

### 5.1 Implementation steps

Based on recommendations from WHO and European Observatory reports (26, 28, 29), the EU Annual Action plan on improving the health of North Macedonia (33), in alignment with the VBHC implementation handbook published by EIT Health (15) and the national health strategies (34, 35), five priority steps have been identified as essential for guiding North Macedonia’s transition toward VBHC. First, public investment in health must increase to reduce the current burden of out-of-pocket payments and align resources with EU benchmarks. Second, primary care should be restructured into multidisciplinary teams incentivized to improve outcomes and manage chronic diseases more effectively. Third, digital infrastructure needs to be strengthened to ensure standardized outcome measurement, benchmarking, and data integration across providers. Fourth, procurement and payment models must be reformed to prioritize long-term value instead of short-term cost savings. Finally, training programs and stakeholder engagement are required to build a culture supportive of VBHC.

A good case example for informing these reforms is North Macedonia’s pay-for-performance scheme, implemented between 2014 and 2023. While it aimed to incentivize efficiency, the program primarily rewarded service volume rather than patient outcomes. Its discontinuation in 2024 highlighted important lessons for future reforms: performance incentives must be linked to validated outcome indicators, supported by robust data systems, and carefully designed to avoid unintended consequences. These insights provide valuable guidance for shaping the next phase of reforms and ensuring that implementation steps remain aligned with the EIT Health Implementation Matrix, which emphasizes “recording,” “comparing,” and “improving” outcomes as the foundation for a sustainable transition toward VBHC.

## 5 Conclusion

The healthcare system in North Macedonia, as in many other European countries, is undergoing continuous transformation. To fully align with the principles of VBHC, future reforms must remain adaptable, patient-centered, and focused on enhancing both efficiency and outcomes. Building on successful VBHC models across Europe, key priorities should include the systematic training and education, the establishment of integrated practice units, the use of gap analyses to identify and address system weaknesses, and the continuous refinement of services. By pursuing these measures, North Macedonia can modernize its healthcare system, enhance the quality of care, and ultimately achieve better health outcomes for its population.
